# The distinct morphological phenotypes of Southeast Asian aborigines are shaped by novel mechanisms for adaptation to tropical rainforests

**DOI:** 10.1093/nsr/nwab072

**Published:** 2021-04-27

**Authors:** Xiaoming Zhang, Qi Liu, Hui Zhang, Shilei Zhao, Jiahui Huang, Tuot Sovannary, Long Bunnath, Hong Seang Aun, Ham Samnom, Bing Su, Hua Chen

**Affiliations:** State Key Laboratory of Genetic Resources and Evolution, Kunming Institute of Zoology, Chinese Academy of Sciences, Kunming 650223, China; Center for Excellence in Animal Evolution and Genetics, Chinese Academy of Sciences, Kunming 650223, China; CAS Key Laboratory of Genomic and Precision Medicine, Beijing Institute of Genomics, Chinese Academy of Sciences, Beijing 100101, China; China National Center for Bioinformation, Beijing 100101, China; School of Future Technology and Sino-Danish College, University of Chinese Academy of Sciences, Beijing 100049, China; State Key Laboratory of Genetic Resources and Evolution, Kunming Institute of Zoology, Chinese Academy of Sciences, Kunming 650223, China; Center for Excellence in Animal Evolution and Genetics, Chinese Academy of Sciences, Kunming 650223, China; CAS Key Laboratory of Genomic and Precision Medicine, Beijing Institute of Genomics, Chinese Academy of Sciences, Beijing 100101, China; China National Center for Bioinformation, Beijing 100101, China; School of Future Technology and Sino-Danish College, University of Chinese Academy of Sciences, Beijing 100049, China; State Key Laboratory of Genetic Resources and Evolution, Kunming Institute of Zoology, Chinese Academy of Sciences, Kunming 650223, China; Kunming College of Life Science, University of Chinese Academy of Sciences, Beijing 100101, China; Department of Geography and Land Management, Royal University of Phnom Penh, Phnom Penh 12000, Cambodia; Department of Geography and Land Management, Royal University of Phnom Penh, Phnom Penh 12000, Cambodia; Department of Geography and Land Management, Royal University of Phnom Penh, Phnom Penh 12000, Cambodia; Capacity Development Facilitator for Handicap International Federation and Freelance Research, Battambang 02358, Cambodia; State Key Laboratory of Genetic Resources and Evolution, Kunming Institute of Zoology, Chinese Academy of Sciences, Kunming 650223, China; Center for Excellence in Animal Evolution and Genetics, Chinese Academy of Sciences, Kunming 650223, China; CAS Key Laboratory of Genomic and Precision Medicine, Beijing Institute of Genomics, Chinese Academy of Sciences, Beijing 100101, China; China National Center for Bioinformation, Beijing 100101, China; School of Future Technology and Sino-Danish College, University of Chinese Academy of Sciences, Beijing 100049, China; Center for Excellence in Animal Evolution and Genetics, Chinese Academy of Sciences, Kunming 650223, China

**Keywords:** Cambodian aborigines, genomic polymorphism, positive selection, parallel evolution, environmental adaptation, morphogenesis

## Abstract

Southeast Asian aborigines, the hunter-gatherer populations living in tropical rainforests, exhibit distinct morphological phenotypes, including short stature, dark skin, curly hair and a wide and snub nose. The underlying genetic architecture and evolutionary mechanism of these phenotypes remain a long-term mystery. We conducted whole genome deep sequencing of 81 Cambodian aborigines from eight ethnic groups. Through a genome-wide scan of selective sweeps, we discovered key genes harboring Cambodian-enriched mutations that may contribute to their phenotypes, including two hair morphogenesis genes (*TCHH* and *TCHHL1*), one nasal morphology gene (*PAX3*) and a set of genes (such as *ENTPD1-AS1*) associated with short stature. The identified new genes and novel mutations suggest an independent origin of the distinct phenotypes in Cambodian aborigines through parallel evolution, refuting the long-standing argument on the common ancestry of these phenotypes among the worldwide rainforest hunter-gatherers. Notably, our discovery reveals that various types of molecular mechanisms, including antisense transcription and epigenetic regulation, contribute to human morphogenesis, providing novel insights into the genetics of human environmental adaptation.

## INTRODUCTION

Modern humans have demonstrated divergent morphological traits among ethnic groups since their ancestors migrated out of Africa and colonized the world more than 120 kya (thousand years) ago. However, the genetic architecture underlying most of these phenotypic divergences remains elusive. Whether these phenotypes are attributed to adaptation to local environments, and how evolutionary forces drive the morphogenesis, have been long-standing essential questions in physical anthropology and human evolutionary genetics [1,2]. In recent years, population analysis of genomic data has served as a powerful approach to addressing these questions [3–9].

Worldwide, ∼50 million people live in tropical rainforests (http://www.srl.caltech.edu/personnel/krubal/rainforest/Edit560s6/www/people.html). Rainforests are hot, humid and have limited food. It was hypothesized that people living in the rainforests, usually the hunter-gatherer groups, developed a series of anthropological characteristics to adapt to the local environment, including a short body stature, dark skin pigmentation, a wide and snub nose, and curly hair, sometimes referred to as Negrito or Pygmy phenotypes in the literature [10]. These small-statured populations mainly inhabit the relatively isolated areas of Southeast Asia, Papua New Guinea and the Andaman Islands, while others live in Africa [10]. Small bodies require less food, generate less heat and are easier to move through trees, traits which are presumably adaptive for living in the rainforest [11]. The diversity of nose shapes across human populations has been driven by local adaptation to climate [12]. Wider noses are more adaptive to warm-humid climates, while narrower noses are more adaptive to cold-dry climates as they efficiently warm and humidify inhaled air. Similarly, curly hair is believed to be beneficial for high temperatures as it facilitates the evaporation of sweat and scalp cooling [13,14]. In addition, other life history variables, such as earlier reproduction to compensate for short lifespans, have also been proposed to explain the adaptation to rain forest environments [15].

Besides the small-statured populations in Island Southeast Asia (ISEA), there are also records of similar populations living in Mainland Southeast Asia (MSEA), such as the Taron tribe in the remote region of Mt. Hkakabo Razi of Myanmar [16], and the Maniq people living in southern Thailand [17], suggesting that these groups were historically widespread in SEA. The pre-Neolithic populations of SEA were later replaced or assimilated by the expansion of East Asian (EA) populations, beginning ∼5000 years ago [18], leading to the current scattered distribution in the rural areas of ISEA [19].

The Cambodian aborigines are called ‘Khmer Loeu’ or ‘Highland’ Khmers. There are 17–21 separate indigenous ethnic groups living in the rainforest highlands of remote northeastern provinces, including Ratanakiri, Mondulkiri and Stung Treng. They have historically been hunter-gatherers (foraging, hunting, fishing and gathering), and since ∼1950 have switched to swidden agriculture practice with seasonal hunting and gathering [20]. They speak Austro-Asiatic languages and make up ∼1.34% of the entire population of Cambodia (General Population Census of Cambodia 2008). Our previous mitochondrial genome analyses of these Cambodian aborigines demonstrated that they harbor population-specific and ancient matrilineal lineages dating ∼60–70 kya, an indication of ancient settlement and long-term *in situ* isolation [21]. The Cambodian aborigines are dark skinned with short stature and they have broad and snub noses, and these morphological characteristics are typical of the small-statured groups [6,22].

There are only a handful of studies on the genetic basis of small-statured phenotypes. Through comparison with their neighboring agriculturalists, 16 genomic regions were identified that were associated with small body size and showed a signature of polygenic adaptation in the Batwa pygmy hunter-gatherers of Uganda [23]. Genes and gene sets involved in muscle development, bone synthesis, immunity, reproduction, cell signaling and development, and energy metabolism were reported as the targets of positive selection in the Biaka from the Central African Republic [24]. In addition, a study on the aboriginal population living on the Flores island of Indonesia found that multiple height-related loci are significantly enriched by population differentiation [6]. However, all these studies only focused on height or body size; the evolution of other morphological phenotypes and the underlying genetic mechanism remain poorly understood.

In this study, to understand the genetic basis of the characteristic morphologies of aboriginal Southeast Asians, we conducted whole-genome sequencing (WGS) of 81 Cambodians from eight diverse ethnic groups, and we identified a set of genes showing strong signals of Darwinian positive selection which may contribute to these morphological phenotypes.

## RESULTS

### Cambodian aborigines represent Southeast Asian small-statured groups

**Figure 1.**
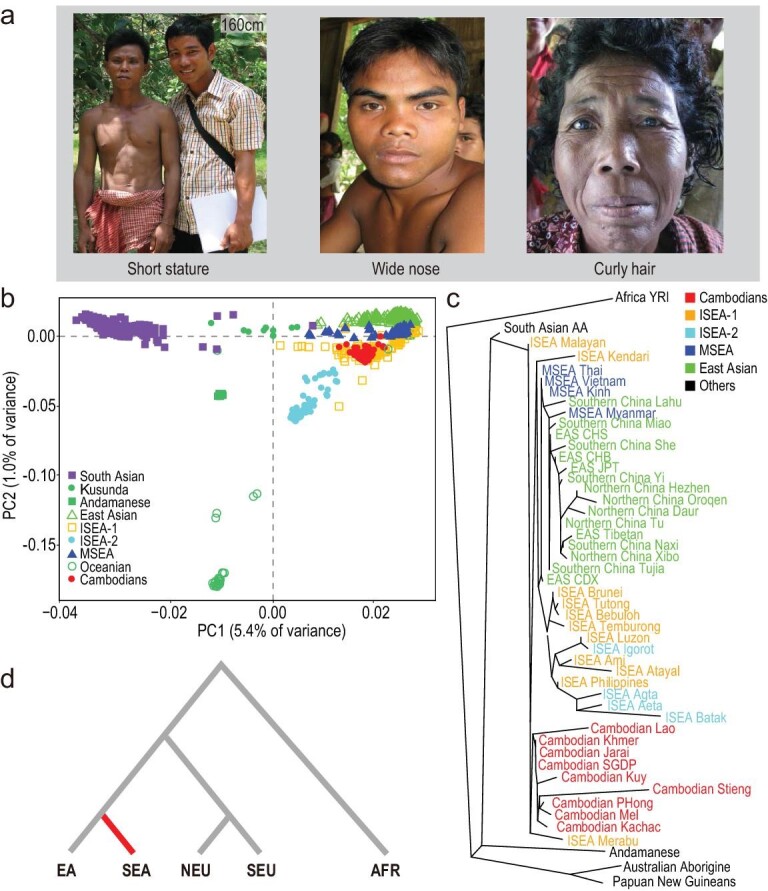
Morphological characters and population affinity of the Cambodian aborigines, and illustration of the scheme for detecting population-specific selection. (a) Morphological characters of the Cambodian aborigines. The Khmer male with a height of 160.0 cm is taller than the Cambodian aboriginal male in the photograph. (b) Principal component analysis (PCA) showing relationships among Asian populations. (c) Tree of Asian populations demonstrating an ancient origin of the Cambodian aborigines and its close proximity to the ISEA populations. Information about the included populations is provided in Supplementary Table S2. (d) The T statistic is constructed to identify population-specific selective signals with a population tree of five continental populations, including Africans (AFR), northern Europeans (NEU), southern Europeans (SEU), East Asians (EA) and Southeast Asians (SEA, the Cambodian aborigines). Abbreviations: MSEA, Mainland Southeast Asian; ISEA-1, Island Southeast Asian; ISEA-2, small-statured ISEA populations.

We investigated the phenotypes by working on the Cambodian aboriginal populations, who were originally hunter-gatherers living in the rainforests of SEA and who show the typical phenotypes of small-statured groups, such as short stature, a wide and snub nose, curly hair and dark skin (Fig. 1a). For example, in the Ratanakiri/Mondulkiri regions where these aboriginal groups mainly dwell, the average adult female height is only 148.7 cm, which is significantly lower than the national average of 152.6 cm (Cambodia Official Demographic and Health Survey, 2000), and much shorter than other continental populations (e.g. the average height of Chinese females is 158.0 cm) (https://worldpopulationreview.com/country-rankings/average-height-by-country).

We selected 81 samples comprised of seven Cambodian aboriginal ethnic groups (Jarai, Kachac, Kuy, Lao, Mel, Phnong and Stieng) and one Khmer population (Supplementary Table S1) to conduct deep whole-genome DNA sequencing (average of 30 × coverage). Principal component analysis (PCA) suggests that, compared to other MSEA and EA populations, Cambodian aborigines are genetically closer to a cluster of ISEA populations (Fig. 1b). This result is consistent with the results of population structure analysis where Cambodian aborigines contain less ancestry from MSEA but more ancestry from ISEA (Supplementary Fig. S1). TreeMix analysis indicates that the Cambodian aborigines are located near the root position of the cluster grouping of all eastern Asian populations, and they separate early from EA and other SEA populations (Fig. 1c). These results thus indicate that the Cambodian aborigines likely represent the descendants of the early modern human settlers in eastern Asia [21].

### Adaptive signals identified in the genomes of the Cambodian aborigines

We performed a genome-wide scan to detect signals of selective sweeps in the Cambodian aborigines using a T-statistic (Fig. 1d; see Methods for details). After removing 27 individuals with cryptic relatedness, the remaining 54 unrelated Cambodian aborigines (identity by descent (IBD) score <0.125) were included in the following analyses. As a reference, we included the published genome data of four continental populations (Africans, northern Europeans, southern Europeans and Han Chinese) (Fig. 1d and Supplementary Table S2; see Methods section for details). The T-statistic is an extension of *F_st_* or the population branch statistics (PBS) from only two or three populations to >3 populations to identify population-specific signals of selection using single-locus allele frequency differentiation (Fig. 1d) (see Methods section for details). In total, we identified 34 013 Cambodian-enriched single nucleotide polymorphisms (SNPs) by the T statistics, representing the top 1‰ of the genome-wide empirical distribution.

To associate the Cambodian-enriched SNPs with genes, the highest one-SNP T statistic from both the coding region and 20 kb upstream of each gene was adopted to represent the gene-level T value. We assessed the significance of each gene by comparing it with the empirical distribution of T from genes with similar sizes (see Methods section for details). We identified 1187 gene regions with a *P*-value <0.05. Gene set enrichment analysis of the 1187 genes identified multiple pathways significantly enriched with signals of natural selection. In particular, several pathways or gene sets related to human morphological traits, including height, hair, facial morphology and skin pigmentation, are significant at a false discovery rate (FDR) of 0.10 (Supplementary Table S3 and S4), implying that local genetic adaptation might have occurred in the Cambodian aborigines driving the formation of their morphological traits.

### A *TCHH* missense mutation contributes to curly hair of Cambodian aborigines

**Figure 2.**
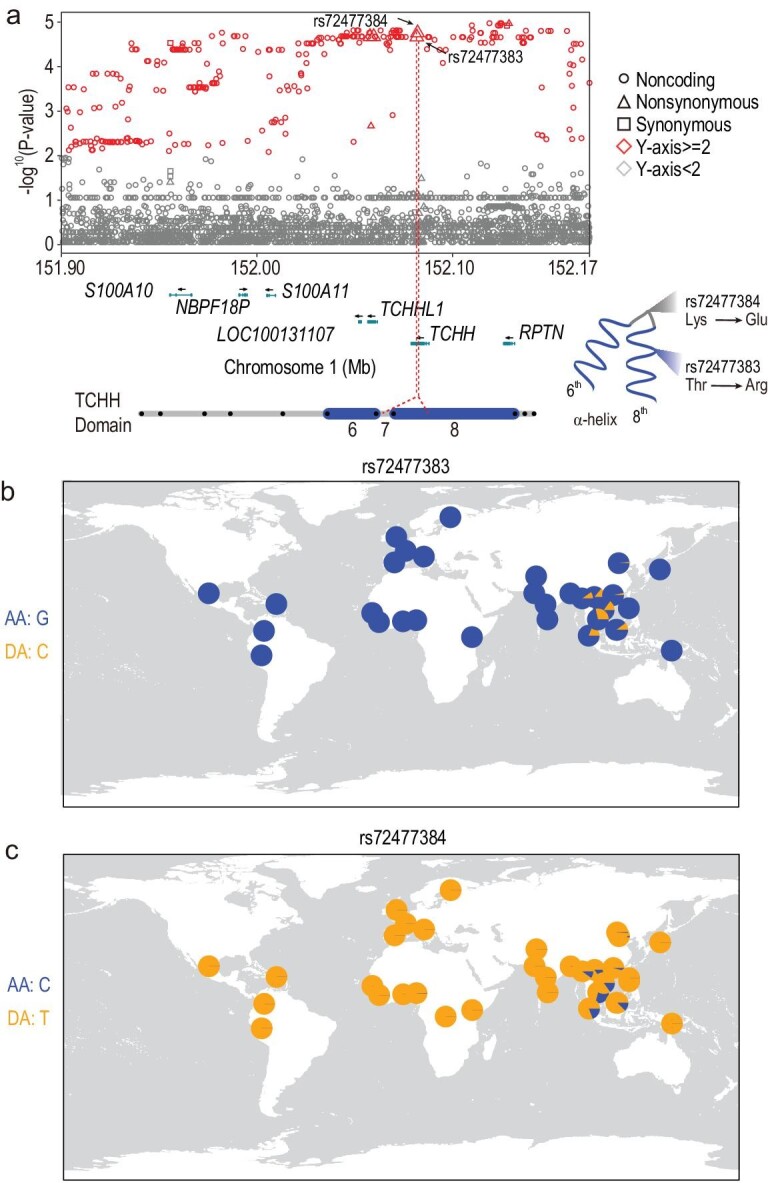
Population genetic signature of positive selection on the *TCHH* gene. (a) Local Manhattan plot of *P*-values of the single-locus T-statistic of SNPs around the *TCHH* gene. Two amino-acid-changing mutations (rs72477383 and rs72477384) showing the most significant signals are located in the 8th and 7th domains of *TCHH* protein. The *P*-values were from the genome-wide empirical distribution, and variants with *P* < 0.01 are colored red. Circles, squares and triangles denote non-coding, synonymous and non-synonymous variants, respectively. The protein structure of *TCHH* was obtained from a previous study [60]. (b and c) The derived allele frequencies of rs72477383 and rs72477384 in world populations. AA, ancestral allele; DA, derived allele.

Hair morphology can be classified into eight types with regard to curliness, with straight hair as type I and the curliest hair as type VIII [25,26]. Anthropological studies showed that Southeast Asians mainly have mildly curly hair (78.00%, type II and III hair) [25], more prevalent than that in East Asians (55.00%). Remarkably, we identify a 270 kb region showing the most significant signal of selective sweep (top 0.1‰) with striking allelic divergences of many SNPs between the Cambodian aborigines (28.70%−32.41%) and the other four continental populations (0.00%−1.85%). This region covers two protein-coding genes, Trichohyalin (*TCHH*, also *THH* or *TRHY*) and Trichohyalin Like 1 (*TCHHL1*) (Fig. 2a), both of which are closely involved in hair morphology. *TCHH* is a major structural protein of the inner root sheath (IRS) cells and medulla layer of the hair follicle [27–29]. The cross-linking between *TCHH* and keratin intermediate filaments (KIFs) is crucial for shaping and mechanical strengthening of the hair shaft [27,30–32]. We found two novel missense variants (rs72477383 and rs72477384) enriched in the Cambodian aborigines. rs72477383 (p.Thr1334Arg) (top 0.1‰) is located in the 8th domain of *TCHH* and acts in the cross-linking between *TCHH* and KIFs [31] (Fig. 2a), therefore might be related to hair morphology change.

Noticeably, rs72477383 is regionally enriched in MSEA and ISEA, but absent in other world populations (Fig. 2b and Supplementary Fig. S3a). The derived allele frequency (DAF) is 31.48% in the Cambodian aborigines, and it is also present in several other SEA populations (10.00%−19.17%) (Fig. 2b). Since SEA populations have a much higher proportion of curly hair than EA populations [25], rs72477383 might contribute to curly hair prevalence in these populations. Another variant rs72477384 (p.Lys1209Glu) is in nearly complete linkage disequilibrium with rs72477383 (r^2 ^= 0.958) with the same distribution pattern in global populations (Fig. 2c). Since the enriched allele of rs72477384 is the ancestral allele, its enrichment in the Cambodian aborigines is likely caused by genetic hitchhiking. The involvement of *TCHH* in hair morphology is further supported by the previous discovery of a rare nonsense mutation (rs201930497) in Europeans leading to uncombable hair syndrome (UHS) with a curly hair phenotype [30]. In addition, another missense variant rs11803731 in *TCHH* was previously reported to be associated with straight hair in Europeans [33].

More intriguingly, we also detected two novel missense variants, rs79690779 (p.Cys789Arg) and rs77167778 (p.Asn167Tyr) in the *TCHHL1* gene, which is in the close vicinity of *TCHH* (17 kb apart) (Supplementary Fig. S2a**)**. *TCHHL1* is restrictedly expressed in the distal inner root sheath of the hair follicle and also plays an important role in hair morphogenesis [34]. These two variants were predicted as functional variants according to the sorting intolerant from tolerant (SIFT) score (<0.05), i.e. 0.038 for rs79690779 and 0.016 for rs77167778 [35]. The geographic distribution of the two *TCHHL1* variants is nearly the same as the two *TCHH* variants due to high linkage disequilibrium (r^2 ^= 1 with rs72477383) (Fig. 2b and c; Supplementary Fig. S2b and c), suggesting that the genomic region covering both *TCHH* and *TCHHL1* is probably the target of selection, and this is further supported by the constructed haplotype networks harboring the four variants (Supplementary Fig. S3a).

Currently, it is still unclear which gene and mutation causes the curly hair phenotype in other small-statured populations of SEA and Africa. Since the four amino-acid-changing variants are SEA-specific, the curly hair phenotype is likely to be of independent origin resulting from parallel evolution in SEA populations.

### Epigenetic regulation of *PAX3* determines the broad and snub nose morphology

**Figure 3.**
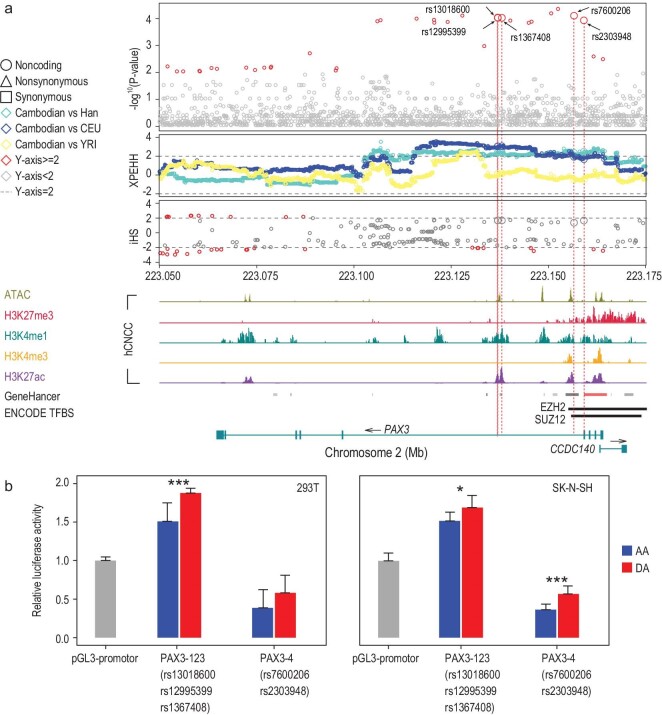
Population genetic signature of positive selection on the *PAX3* gene and results of reporter gene assays demonstrating the enhancer and suppressor activity of multiple variants upstream of *PAX3*. (a) Multiple statistics indicating positive selection on the genomic region harboring the *PAX3* gene. The y axis presents –log_10_ (empirical *P-*value) of the T statistic (the 1st panel), and the normalized XPEHH (the 2nd panel) and iHS values (the 3rd panel). The XPEHH values were calculated by comparing Cambodian aborigines with Han Chinese (Han), Europeans (CEU) and Africans (YRI). Circles, squares and triangles denote non-coding, synonymous and non-synonymous variants, respectively. The dotted lines indicate the cutoff values of 2 and −2. Statistically significant variants are colored red. The profiles of chromatin accessibility (ATAC-seq, olive), H3K27me3 (crimson), H3K4me1 (teal), H3K4me3 (orange) and H3K27ac (purple) chromatin immunoprecipitation (ChIP) were obtained from the published data of human cranial neural crest cells (CNCC) [61]. GeneHancer and ENCODE TFBS annotations are presented. (b) The results of reporter gene assays indicate an increased enhancer activity and a decreased promotor repression of the *PAX3* variants. The three completely linked intronic variants (rs13018600, rs12995399 and rs1367408; r^2 ^= 1) are located in the same predicted enhancer element and were tested together (*PAX3*–123); the other intronic variant (rs7600206), located in the predicted promotor repression element, was tested in a separate assay (*PAX3*–4). The assays were performed using 293T (left panel) and SK-N-SH (right panel) cells. ^*^P < 0.05, ^**^P < 0.01, ^***^P < 0.001.

The shape of the nose and the width of the nasal cavity is thought to reflect climate adaptation when populations move to a new environment [12]. A broad nose has evolved in response to warm-humid climates, though no responsible genes have been reported [12]. We identified a region under selection in the Cambodian aborigines. This region contains the *PAX3* gene that encodes a transcription factor, which is associated with nasion prominence and nose width in Europeans reported by several recent genome-wide association studies (GWASs) [36–38]. The region is among the genome-wide top 0.1‰ of the T statistic, and demonstrates a prominent long-range haplotype caused by strong positive selection with high Cross Population Extended Haplotype Homozogysity (XPEHH) and iHS scores (Fig. 3a). Within this region, there are three completely linked intronic variants (rs13018600, rs12995399 and rs1367408; r^2 ^= 1) showing highly diverged frequencies between the Cambodian aborigines (69.44%) and the other populations (26.92%−33.50% in East Asians, 16.67%−24.30% in Europeans and 3.54%−12.04% in Africans) (Supplementary Fig. S3b).

More importantly, by searching for the histone modification data of human cranial neural crest cells (CNCC) we show that the three variants are located in the H3K27ac and H3K4m1 peaks, an indication of enhancer elements that may regulate *PAX3* expression. Notably, the positional plasticity of pre-migratory CNCC progenitors is essential for the assembly of distinct craniofacial structures [39]. We performed *in vitro* enhancer assays and the results show that the derived haplotype covering the three intronic variants significantly increases enhancer activities compared to the ancestral haplotype in both 293T and SK-N-SH cells (Fig. 3b), supporting a functional role of the variants under selection in the Cambodian aborigines.

In addition, there are two other *PAX3* intronic variants (rs7600206 and rs2303948) among the top 1‰ list, which are 18.5 kb away from the above three variants with strong linkage disequilibrium (r^2 ^= 0.81). Similarly, the haplotype carrying the derived alleles of these two variants is highly enriched in the Cambodian aborigines (63.89%), while much less in the other populations (11.10%–24.50%) (Supplementary Fig. S3c). rs7600206 is located in the H3K27ac and H3K27me3 peak regions of human CNCC, the signal of a promotor repression element that contains the binding site of EZH2. EZH2 is an essential component of the PRC2/EED-EZH2 complex, and a machine generating the H3K27me3 modification (Fig. 3a). Previous research suggested that the Ezh2-dependent poised chromatin (H3K27me3+/H3K4me2+bivalency) organization determines the positional plasticity of the pre-migratory CNCC progenitors, and is essential for the assembly of distinct craniofacial structures [39].

Chromatin profile data in mouse embryos indicate that the promotor/enhancer region of *PAX3* presents H3K27me3+/H3K4me2+bivalency pattern in the pre-migratory CNCC progenitors and *PAX3* is a frontonasal (FNP)-specific positional transcription factor (Supplementary Fig. S4) [39]. To validate the speculated function of the two variants, we performed reporter gene assays and found that the derived allele of rs7600206 has a reduced suppressor activity compared to the ancestral allele (Fig. 3b), consistent with the observed increased enhancer activity of the aforementioned three *PAX3* variants.

Taken together, these results suggest that the selected region located in the intronic region of *PAX3* containing regulatory elements (enhancer and promotor repression elements) may upregulate *PAX3* through EZH2-mediated epigenetic regulation, which may contribute to the nasal morphogenesis change of the Cambodian aborigines. Notably, this is the first reported case that suggests mutations in the epigenetic regulation motifs may play crucial roles in human phenotype evolution.

### Antisense transcription of *ENTPD1-AS1* regulates the short stature of Cambodian aborigines

**Figure 4.**
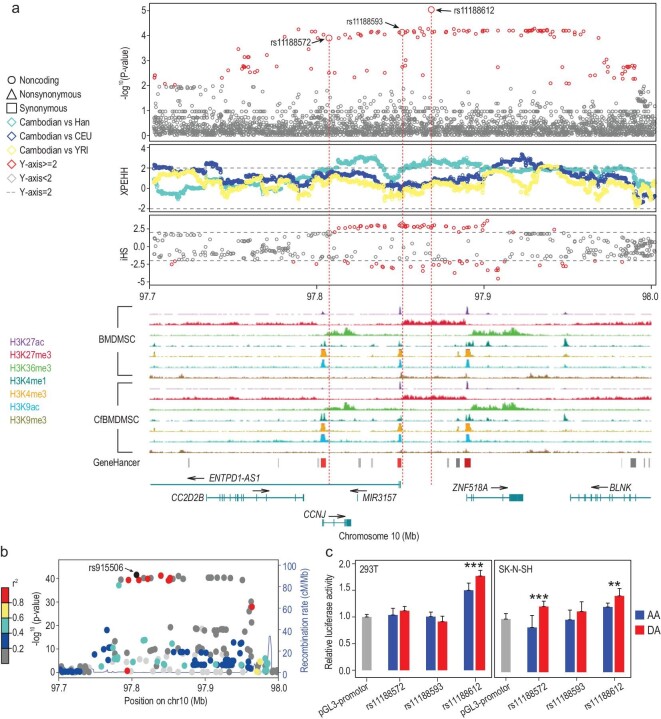
Population genetic signature of positive selection around the *ENTPD1-AS1* gene. (a) Multiple statistics indicating positive selection on the genomic region harboring the *ENTPD1-AS1* gene. The y axis presents the –log_10_ (empirical *P*-value) of the T statistic, the normalized XPEHH and iHS values (the 1st–3rd panels). The epigenetic profiles were obtained from the Roadmap dataset (the 4th and 5th panels) [62]. The statistically significant variants are colored red, and circles, squares and triangles correspond to non-coding, synonymous and non-synonymous mutants, respectively. The dotted lines indicate 2 and −2. (b) Local Manhattan plot showing the association of height with variants around the *ENTPD1-AS1* gene in Europeans (the *P-*values are from the GWAS study of height in Europeans [43]). rs915506 shows the strongest signal. The other SNPs are colored according to linkage disequilibrium with rs915506 in the Cambodian aborigines. (c) The results of enhancer assays indicate an increased enhancer activity of the adaptive allele of rs11188612, while no statistically significant changes were detected in the other two variants. Both 293T and SK-N-SH cells were tested. ^*^P < 0.05, ^**^P < 0.01, ^***^P < 0.001.

The average height of Cambodian males is 160 cm (https://brandongaille.com/list-average-human-male-height-by-country/) and the Cambodian aborigine males should be shorter though the exact value is not available. This height is greater than the small-statured populations in Africa (150.0 cm for males) [40] and ISEA (150.7 cm for males) [41], while much smaller than East Asians (169.5 cm for Chinese males) and Europeans (175.3 cm for English males) [42]. We identified a set of genes with enriched signals of positive selection, and previous GWASs demonstrated significant association of these genes to human height. In particular, one 220 kb region upstream of the *ENTPD1-AS1* gene shows an extremely significant *P*-value (<1‰) of T statistic in the Cambodian aborigines (Fig. 4a). Within this region, 36 out of the 87 top 1‰ SNPs are Asian-specific, with the DAF being around 65.14% in Cambodian aborigines, 44.46% in Han Chinese and nearly absent in Africans and Europeans (<1.00%) (Supplementary Table S5).

In addition to high population differentiation, the iHS and XPEHH statistics also indicate the effect of positive selection on this region in the Cambodian aborigines (Fig. 4a). A long-range haplotype carrying multiple variants with selective signals occurred in multiple SEA populations including Cambodians, Malaysians and CDX (Daic speakers from southwestern China) (Supplementary Fig. S3d). Interestingly, this region covers multiple promoter and enhancer elements harboring the detected variants under selection, which may change the expression regulation of *ENTPD1-AS1* (Fig. 4a).

The GWAS data from the Genetic Investigation of Anthropometric Traits (GIANT) show that this region is strongly associated with height in Europeans [43] (meta *P*-values < 1e-30) (Fig. 4b), and the derived alleles (enriched in the Cambodian aborigines) are associated with decreased height (Supplementary Table S5). Accordingly, the eight variants with meta *P*-values < 1e-30 (absolute effect size around 0.02) are all among the significant variants (top 1%) under positive selection and they are strongly linked with the top 1‰ variants (Supplementary Table S5). Consistently, another GWAS analysis identified SNP markers in this region to be associated with height in East Asians [44].

To validate the speculated function of significant variants, we performed reporter gene assays, and we found that the derived allele of rs11188612 (the most significant mutation in this region) has significantly increased enhancer activity compared to its ancestral allele (Fig. 4c). *ENTPD1-AS1* functions as an antisense RNA of the *ENTPD1/CD39* gene, and it can regulate the transcription of *ENTPD1* through antisense transcription [45]. Furthermore, *ENTPD1* as an ATPase decreases interleukin 1 beta (*IL-1β*) expression [46], a pro-inflammatory cytokine that directly acts on growth plate cartilage and suppresses bone growth [47–49]. We thus speculate that *ENTPD1-AS1* may serve as a major gene for the short stature of Cambodian aborigines and has undergone parallel evolution in SEA. Antisense transcription is known to be an efficient mechanism of rapid evolution in bacteria and mammals [50], and again, our discovery is the first case of phenotype evolution attributed to antisense-mediated gene regulation in humans.

## DISCUSSION

The evolution of human morphological traits is of great interest, however, most of them remain unknown except for a few cases, e.g. pigmentation [51–54]. In this study, we use population genomic approaches to identify genes underlying the distinct morphological phenotypes of the Cambodian aborigines, the hunter-gatherer groups living in MSEA who exhibit short stature, dark skin, curly hair and broad and snub noses. We present multiple lines of evidence to demonstrate that these genes and the putative causal variants are under strong selection and thus provide clues to answering the long-term hypothesis that these phenotypes are shaped by environmental adaptation to tropical rainforests.

Our discovery sheds new light on the evolution of human morphological traits in three aspects. Firstly, the putative causal mutations of curly hair and height, which all act on novel mutations, occurred in Asian or SEA populations. Although the phenotypes are similar among different tropical ethnic groups including African populations, the SEA aborigines likely developed these adaptive traits independently by recruiting new genes and new mutations, as a typical convergent or parallel evolution. Secondly, it is commonly agreed that most of the morphological traits, for example, the facial morphology and the stature/height, are quantitative traits controlled by multiple genes with minor effects, an implication of neutral evolution of these traits. However, in our study the genes show strong signals of positive selection, including a prominent long-range haplotype and strong positive selection.

**Figure 5.**
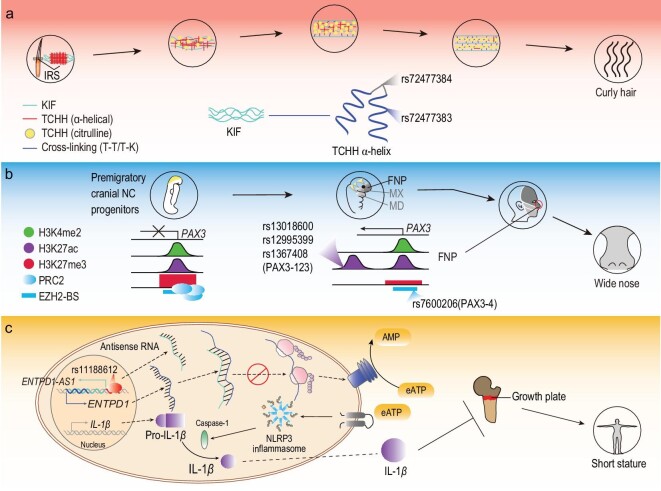
Schematic illustration of the proposed molecular mechanisms of the three genes and mutants contributing to the distinct morphological phenotypes of Cambodian aborigines. (a) The two identified adaptive variants of *TCHH* potentially disrupt the alpha-helix structure of *TCHH* protein, and further destabilize the *TCHH-TCHH* and *TCHH*-KIF cross-linking, which are crucial for the straightness of hair. The figure was adapted from a previously published model [27]. KIF, keratin intermediate filaments; T–T, *TCHH* cross-linked with *TCHH*; T–K, *TCHH* cross-linked with KIF. (b) Adaptive variants upstream of *PAX3* affect nose morphology of the Cambodian aborigines. The mutants rs7600206 and rs2303948 are located in the epigenetic regulation region upstream of *PAX3*, containing the binding site of EZH2, which represses the expression of *PAX3* in pre-migratory CNCCs, while tissue specifically activates it in the FNP area. The other three mutants (rs13018600, rs12995399 and rs1367408) are in the enhancer elements that regulate the *PAX3* expression. The epigenetic regulation of human CNCC positional identity was adapted from Minoux *et al.* [39]. TFBS, transcription factor binding sites; hCNCC, human cranial neural crest cells; FNP, frontonasal; MX, maxillary; MD, mandibular. (c) The putative role of rs11188612 in the antisense transcription of *ENTPD1*-*AS1* and the regulation of short stature. *ENTPD1-AS1* regulates the expression of *ENTPD1* by antisense transcription, which decreases *IL-1β*, and further affects the body stature of Cambodian aborigines through the *ENTPD1*/*IL-1β*/growth plate pathways. BMDMSC, bone marrow derived mesenchymal stem cell; CfBMSMSC, chondrocytes from bone marrow derived mesenchymal stem cell.

Lastly, but most importantly, various novel mechanisms are likely recruited in the adaptive evolution of human morphological traits, and we summarized the putative mechanistic models in Fig. 5. Curly hair of Southeast Asians might be related to the Asian-specific missense mutation rs72477383 of the *TCHH* gene through the cross-linking between *TCHH* and KIFs (Fig. 5a). Mutations in the EZH2-binding sites upstream of *PAX3* may mediate epigenetic regulation of *PAX3* and possibly contribute to nasal morphology (Fig. 5b). *ENTPD1-AS1* may explain the short stature of the Cambodian aborigines through antisense transcription (Fig. 5c). Our study thus provides novel insights into the evolutionary pattern and mechanism of human morphogenesis.

## MATERIALS AND METHODS

### Sample collection and whole-genome DNA sequencing

From the 1054 Cambodian individuals collected in our previous studies, we randomly sampled 81 Cambodian aborigines for deep WGS from eight ethnic groups, including seven aboriginal populations and one Khmer population, from three mountainous provinces in northeastern Cambodia. We generated high-coverage (average ∼30×) WGS data from genomic DNA for the 81 Cambodian aborigine samples (Supplementary Table S1). Additional genomic data were collected from the following public sources: the 1000 Genomes Project Phase 3 (KGPp3), the Estonian Biocentre Human Genome Diversity Panel (EGDP), the Simons Genome Diversity Project (SGDP), the Singapore Sequencing Indian Project (SSIP), Singapore Sequencing Malay Project (SSMP), and the Tibetan, Andamanese, Malay and CAS-PMI project. This comprised a total of 3515 samples from 231 global populations (Supplementary Table S2). Details are provided in the supplementary data.

### Population analyses

To explore population relationship between the Cambodian aborigines and other global populations, PCA was performed using smartpca in EIGENSOFT-v6.1.4 [55]. To infer the phylogenetic relationship of populations, a maximum likelihood tree was constructed by using the TreeMix program [56]. We developed a statistical method to identify population-specific signals of selection with a T statistic, which uses single-locus allele frequency differentiation and is an extension of *F_st_* or PBS [57], for two or three populations to multiple populations. Details are provided in Supplementary Data.

### Gene function analysis

The highest single-SNP T statistic from each gene region (including the coding region and 20 kb upstream of the gene) was adopted to represent the gene-level T value. To correct for the potential bias by gene length difference, we assessed the significance of each gene by comparing the gene-level T with the empirical distribution of T from genes with similar sizes [58]. Functional enrichment for candidate gene regions was performed using the annotation tool KOBAS 3.0 (Supplementary Tables S3 and S4). Association summary statistics for height were downloaded from the GIANT consortium. Haplotype networks were constructed with the median joining method [59] and visualized using NETWORK v10 Software (https://www.fluxus-engineering.com/). Geographic distribution of variant frequencies in world populations were generated with self-created R scripts. Details are provided in the supplementary data.

### Reporter gene assays

We chose *PAX3*–123 (rs13018600, rs12995399 and rs1367408), *PAX3*–4 (rs7600206), *ENTPD1*–1 (rs11188572), *ENTPD1*–2 (rs11188593) and *ENTPD1*–3 (rs11188612) to test their potential effect on enhancer activity by luciferase reporter assays. The mean values of three independent experiments were used. Each independent experiment has three replicates so that nine data points were generated for each allele of the tested SNPs. Details are provided in the supplementary data.

## Supplementary Material

nwab072_Supplemental_Files

## Data Availability

All data reported in this study have been deposited in the Genome Sequence Archive in the National Genomics Data Center, Beijing Institute of Genomics (China National Center for Bioinformation), Chinese Academy of Sciences, and the data are publicly accessible at https://bigd.big.ac.cn/gsa under the accession number of HRA000316.
