# Supplementary material for: The distinct morphological phenotypes of Southeast Asian aborigines are shaped by novel mechanisms for adaptation to tropical rainforests
Source: Natl Sci Rev. 2021 Apr 27;9(3):nwab072. doi: 10.1093/nsr/nwab072 (PMC8970429; doi:10.1093/nsr/nwab072)

**Supplementary Figure S1. Population structure inferred by clustering analysis.**  The analysis was performed on the WGS data using ADMIXTURE with the number of clusters (K) ranging from 8 to 12. Each individual is represented by a single vertical bar colored in proportion to their estimated ancestry within each cluster. Each color represents one putative ancestral cluster. The zoom-in panel shows the pattern of regional populations (K = 12) from Southeast Asia, Oceania and southern China. Abbreviation: SA-South Asian; MSEA-Mainland Southeast Asian; ISEA-Islands Southeast Asian; WGS-whole genome sequencing; KGPp3-1,000 Genomes Project Phase 3.


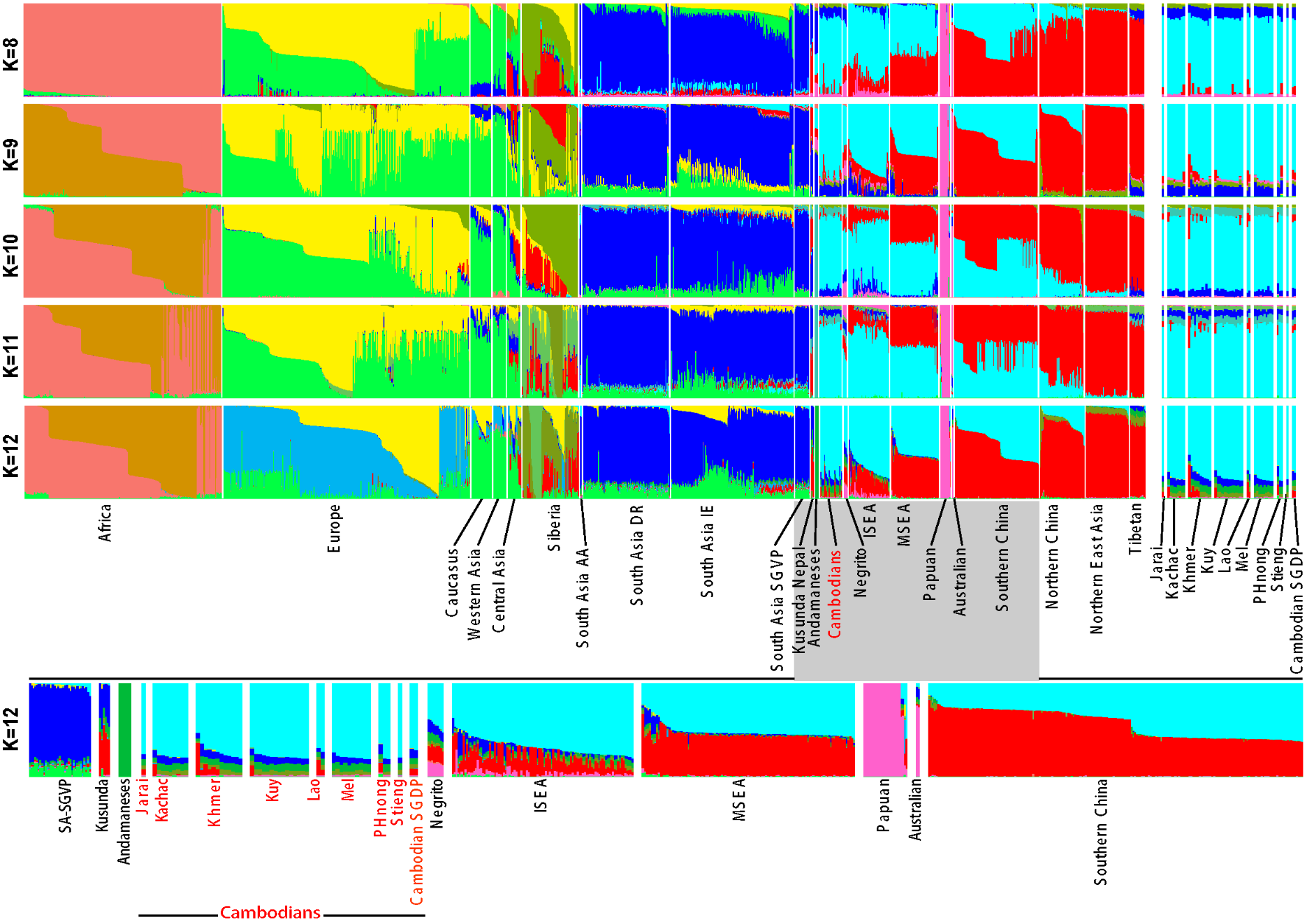


**Supplementary Figure S2. Genetic polymorphism pattern indicates positive selection on the *TCHHL1* gene.** (**A**) The plot of –log_10_ (P) values of T statistic of variants near the *TCHHL1* gene in Cambodian aborigine genomes. The P values are from the genome-wide empirical distribution. Variants with –log_10_(P) >2 are marked red. Circles, squares, and triangles denote noncoding, synonymous, and nonsynonymous variants, respectively. (**B** and **C**) The geographic distribution of the derived-allele frequencies of rs77167778 and rs79690779 in world populations. AA-ancestral allele, DA-derived allele.


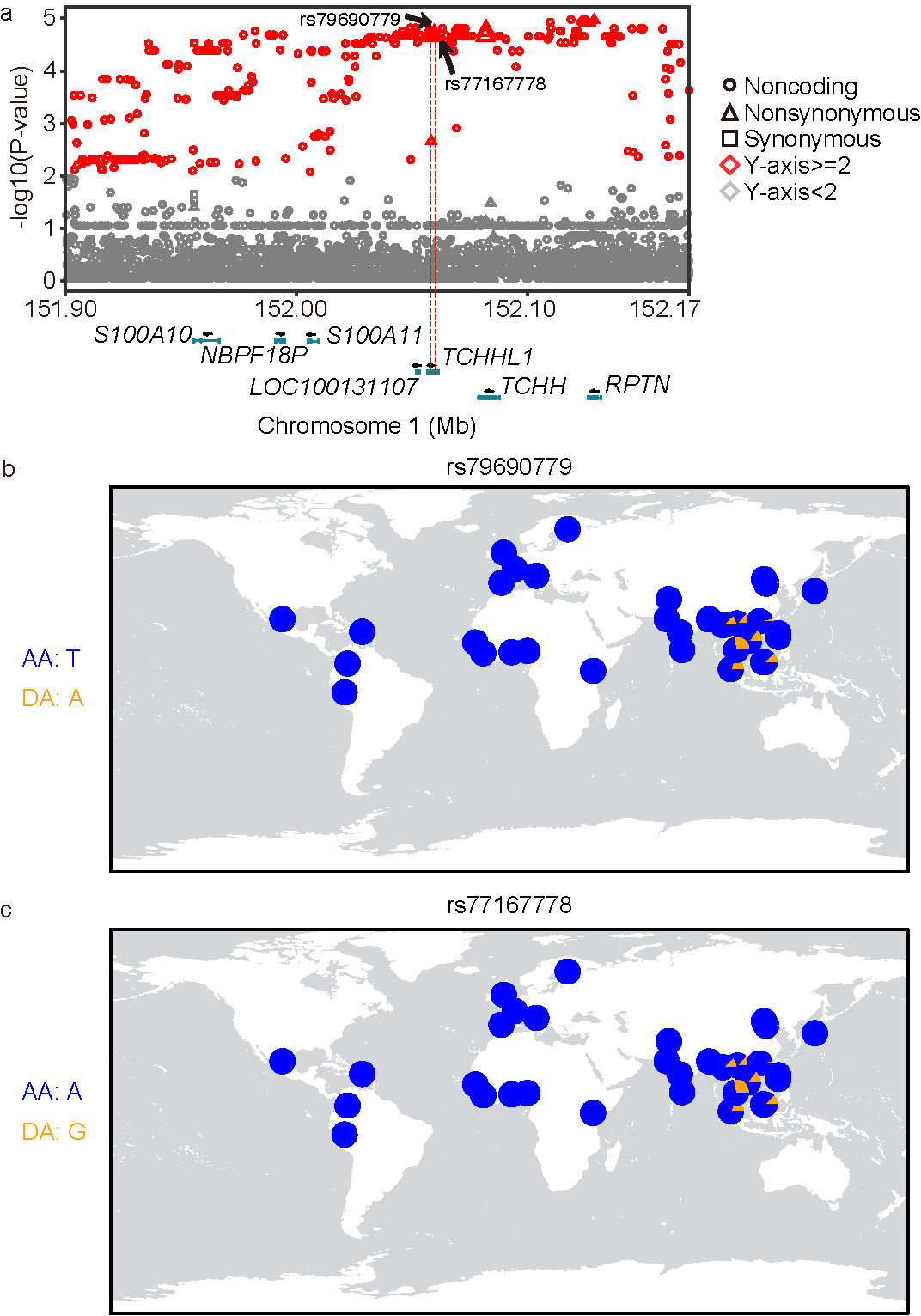


**Supplementary Figure S3. Haplotype networks of genomic regions containing *TCHH & TCHHL1*, *PAX3*, and *ENTPD1-AS1*.** The haplotype networks were constructed with the Median-joining method. The sizes of nodes are proportional to the counts of the haplotypes. Proportions of the population origins of the haplotypes are displayed as pie charts within nodes. Turquoise and purple panels indicate the haplotypes carrying the derived alleles (turquoise) or the ancestral alleles (purple). The shadowed ellipse marked the Southeast Asian- or the Cambodian aborigine –specific haplotypes. (A) The 24.5kb gene region covering *TCHH* & *TCHHL1*. (B) The 17.8kb gene region covering *PAX3*. (C) The 2.5kb gene region covering *PAX3*. (D) The 4.9kb gene region covering *ENTPD1-AS1*. YRI-Africans, TSI-southern Europeans, CEU-northern Europeans, Han-Han Chinese, CDX-The Daic speaker from southwestern China.


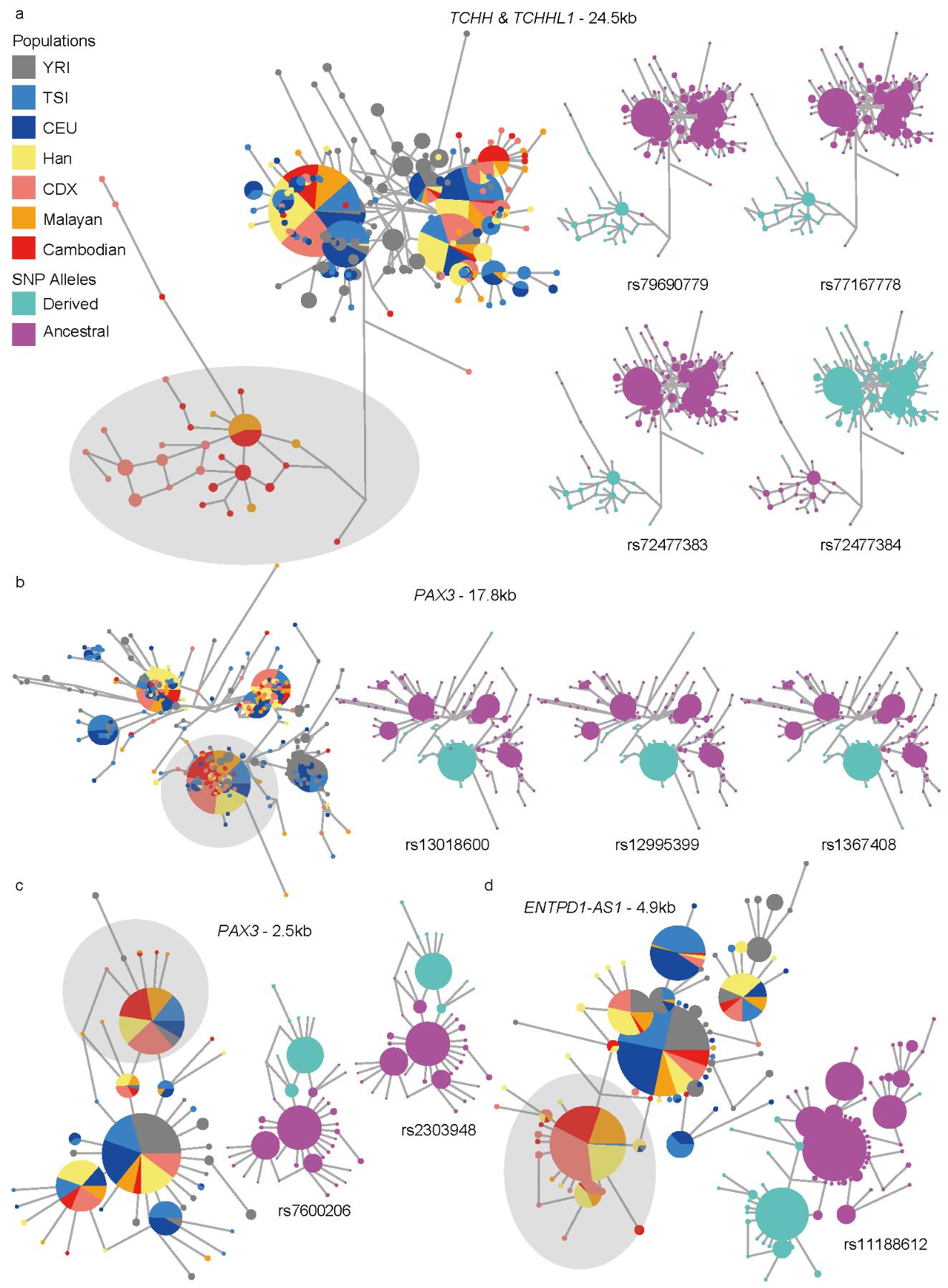


**Supplementary Figure S4. Chromatin annotation of the *PAX3* gene region in mouse CNC cells during the post-migratory and pre-migratory stages.** Chromatin accessibility (ATAC-seq, khaki), H3K27me3 (red), H3K4me2 (cyan), H3K27ac (blue), and H3K27me3/H3K4me2 double ChIP (orange) profiles are shown in E8.5 premigratory CNC progenitors and E10.5 NC cells of FNP, MX, and MD processes. All data were retrieved from Minoux et al (38). The shaded box indicates the enhancer regions of *PAX3*. CNC, cranial neural crest; FNP, frontonasal; MX, maxillary; MD, mandibular.


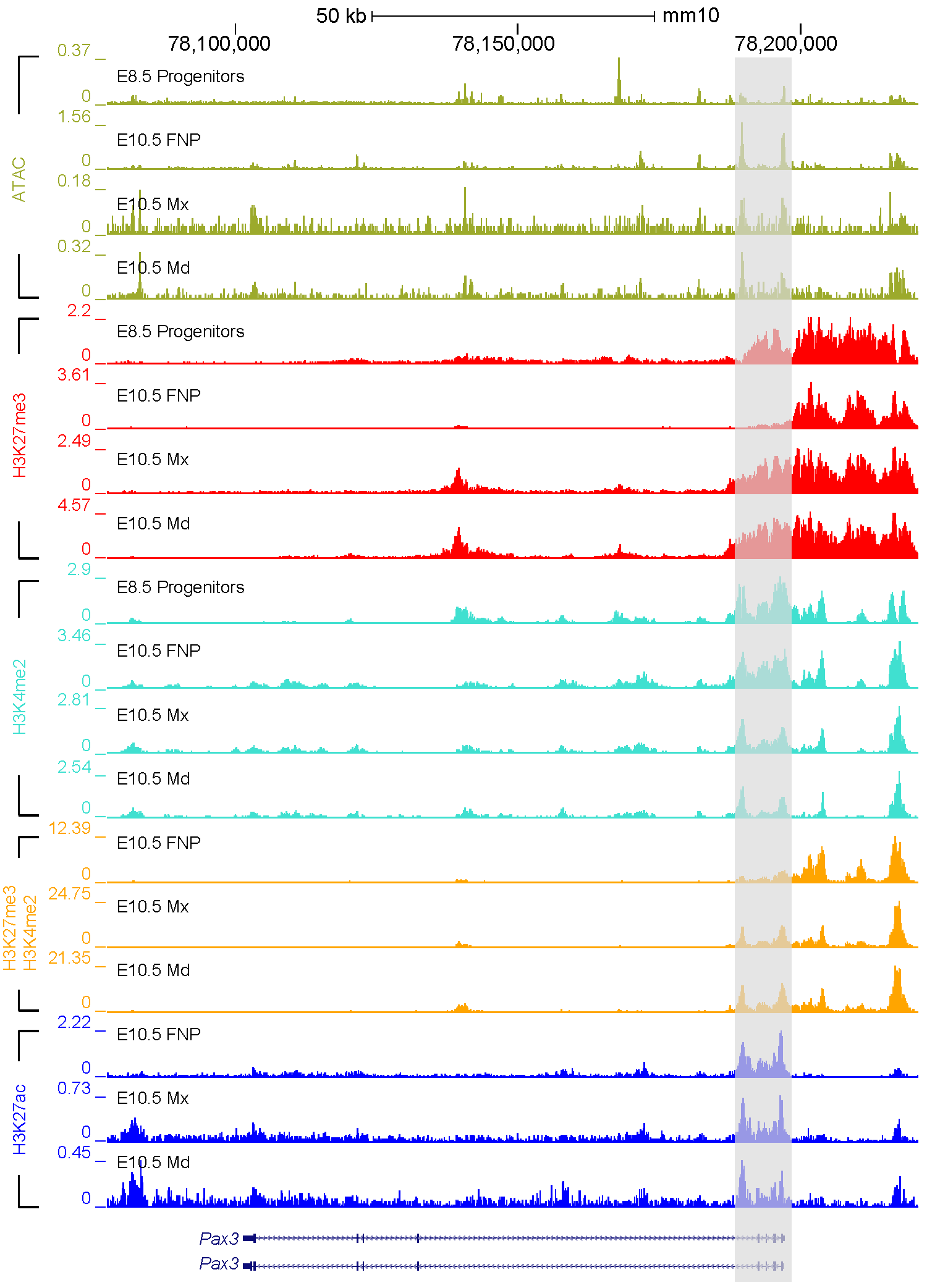

Supplement: nwab072_Supplemental_Files [file nwab072_supplemental_files.zip › Supplementary_Figures_Revision.docx]
