# Supplementary material for: The distinct morphological phenotypes of Southeast Asian aborigines are shaped by novel mechanisms for adaptation to tropical rainforests
Source: Natl Sci Rev. 2021 Apr 27;9(3):nwab072. doi: 10.1093/nsr/nwab072 (PMC8970429; doi:10.1093/nsr/nwab072)
Supplement: nwab072_Supplemental_Files [file nwab072_supplemental_files.zip › Supplementary_Methods_Final_Version.docx]

**Materials and Methods**

**Sample Collection**

From the 1,054 Cambodian individuals collected in our previous studies [1, 2], we randomly selected 81 Cambodian aborigines for deep whole genome sequencing that comprise 8 ethnic groups, including 7 aboriginal populations and 1 Khmer population from three mountainous provinces in northeastern Cambodia. The detailed information of the samples can be seen in Supplementary Table S1. Ethics approval was obtained from Internal Review Board of Kunming Institute of Zoology, Chinese Academy of Sciences and the Royal University of Phnom Penh, Cambodia. Informed consents were obtained from all the participants that were written in both Khmer and English languages.

**Whole genome DNA sequencing**

We generated high-coverage (average ~30 ×) whole-genome sequencing (WGS) data from genomic DNA for the 81 Cambodian aborigine samples from 8 ethnic groups (see Supplementary Table S1). Sequencing libraries were prepared and sequenced as 150bp paired-end reads on Illumina Hiseq X Ten platform. Adapter were trimmed from raw reads using AdapterRemoval [3]. The cleaned reads were aligned to the human reference genome (GRCh37) using the BWA-MEM algorithm [4]. PCR duplicates were removed using Picard (http://broadinstitute.github.io/picard/). Local realignment around insertions and deletions (INDELs), and base quality score recalibration were performed with GATK (https://software.broadinstitute.org/gatk/) following the recommended settings. A total of 15,956,274 raw variants including 13,104,020 SNVs and 2,852,254 indels were obtained. After further applying the variant quality score recalibration with GATK, 10,417,988 bi-allelic SNVs were remained. The genomic data of a final set of 54 Cambodians (see Supplementary Table S1) were used for further analysis, after filtering out individuals with first- to third-degree cryptic relatives according to kinship analysis with KING [5].

**Integrating Cambodian WGS with global population datasets**

Additional personal genomic data were collected from the following public sources: the 1000 Genomes Project Phase 3 (KGPp3) [6], EGDP [7], SGDP [8], SSIP [9], SSMP [10], Tibetan [11], Andamanese [12], Malaysian aborigines [13], and the CAS-PMI project [14] , comprising a total of 3,515 individuals from 231 world populations (see Supplementary Table S2 for details).

For the analysis of population structure and genetic relationship, we merged the single nucleotide polymorphisms (SNPs) of Cambodian genomes and other global populations to construct an intersection of SNPs. For selection analysis, we merged the SNPs of Cambodian genomes and the four reference populations from the KGPp3 and the CAS-PMI project to construct a union of SNPs. For each population, the variants of that population in the union SNP set but not in the raw variants were extracted from the BAM file using self-created scripts. The genotypes of these loci are the homozygote of allele with the highest coverage (i.e. 95% coverage and read depth range from 1/3 to 2.5 of mean depth of twenty samples), which were then merged into raw dataset of that population. This operation can efficiently retain population-specific variants in order to identify population-specific selection signals.

**Population structure and phylogenetic analysis**

To explore population relationship between the Cambodian aborigines and other global populations, principal component analysis (PCA) was performed using smartpca of EIGENSOFT-v6.1.4 [15] with 1,467 individuals and 424,071 autosomal bi-allelic SNPs that have missing call rates of less than 10% in each populations. To infer the phylogenetic relationship of the populations, a maximum likelihood tree was constructed by using the TreeMix program [16] based on 937 individuals and 3,064,062 autosomal bi-allelic SNPs with no missing data. Migration events were sequentially interrogated using the argument of –m, and TreeMix accounted for LD by grouping SNPs in a window size (-k) of 500, approximately corresponding to 10Mb. For all TreeMix analyses, we set the African (YRI) population as the outgroup. We used the software ADMIXTURE [15] for population structure analysis. To avoid the effects of linkage disequilibrium (LD), we pruned the dataset using PLINK v1.9 (67) employing a window of 200 SNPs with sliding window of 25 SNPs and an r2 threshold of 0.4(indep pairwise 200 25 0.4). After pruning, 331,059 SNPs and 2,762 individuals were left for further analysis. Then we ran ADMIXTURE with random seed, exploring the number of clusters (K) ranging from 2 to 12.

**Detection of population-specific positive selection**

We developed a statistical method to identify population-specific signals of selection with a T statistic, which uses single-locus allele frequency differentiation and is an extension of *Fst*or the population branch statistic (PBS) [17] from two or three populations to multiple populations. Let *S* be the set of external nodes of an evolutionary population tree, and *L* be the set of branches of the tree. The T statistic is derived as follows. For any couple of populations from S, , there is a unique route on the tree connecting and , which includes branch lengths shared by the paths from the root to nodes and , denoted as . Let be the length of branch k, for any two populations of the evolutionary tree, is approximately equal to twice of the population divergence time T in units of *2N* (assuming the two populations are of the same size *N* [18]).

(1)

For four or more populations, we have equations following Eqn. (1) for all population pairs, which together form an overdetermined system of equations. The equation system can be solved by the least square method, and for any locus in the genome, we can obtain a set of values for all branches of the population tree. The branches leading to external population nodes (tree tips) can be an indicator of the evolution rates in those populations, serving as a summary statistic for detecting population-specific natural selection. Similarly, the internal branches of the population tree are indicators of evolution rates in ancestral populations, and are informative to detect selection in those ancestral populations. We use genome-wide empirical distribution of to assess the significance level of a single SNP locus. The SNPs with the upper 0.1% limits of the distribution on each branch were considered as the signatures of positive selection, which correspond to a significant level of 0.001.

When solving Eqn. (1) with the least square method, we have additional restrictions that all the variables . If two external population nodes are with , then the value of right part of Eqn. (1) is infinity. In order to deal with the non-trivial situation, we set the up bound of to be a sufficiently large value, e.g., 5.

Here we used five geographic populations including Cambodian aborigines (Southeast Asian, SEA), and Han Chinese from CAS-PMI project (East Asian, EA), CEU (North Europeans, NEU), TSI (South Europeans, SEU) and YRI (African, AFR) from the KGPp3 to identify Cambodian-specific signals of selection. There are seven variables *T*1-*T*7 on the phylogenetic tree, and ten constraint conditions among these variables according to equation (1) (see the schematic illustration). For example, from EA and SEA, we obtained the constraint . All these constraints make up the system of equations (2), which is overdetermined equations with seven variables and ten constraints. We applied MATLAB function lsqlin to obtain the least square fitting of (2).

(2)

We also performed haplotype-based tests to confirm the identified signatures of selection (i.e. iHS [19] and XP-EHH [20]). Beagle 5.0 [21] was used to infer the haplotypes from genotypes of the studied populations. The 1,000 Genomes Project ancestral alignment file was used to identify ancestral alleles of SNPs [6] (<ftp://ftp.1000genomes.ebi.ac.uk/vol1/ftp/phase1/analysis_results/supporting/ancestral_alignments/>). The Selscan software [22] was used to calculate iHS and XP-EHH values with the default parameters.

**Gene function analysis**

To associate the Cambodian-enriched SNPs with genes, we firstly determine the range of a gene region, which includes the coding region (from the starting position to the ending position of all transcripts) and 20kb upstream of the gene. We adopted the highest single-SNP within the gene region to represent the gene-level value. To achieve the gene-level p value and correct for the bias caused by difference in gene lengths, we used the approach by Daub et al. (2013) [23]. In total, 1,187 genes were identified as significant at 0.05 level. Functional enrichment for candidate gene regions was performed using the annotation tool KOBAS 3.0 [24] with multiple pathway databases (i.e. KEGG, Reactome, BioCyc and PANTHER), disease databases (i.e. OMIM, KEGG Disease and NHGRI GWAS Catalog), and Gene Ontology databases (see Supplementary Table S3 and Table S4). FDR correction for multiple tests in enrichment analysis was performed using the *fdrtool* function from the R package fdrtool [25]. H3K4me3, H3K4me1, H3K27me3, H3K27ac and ATAC-seq signal tracks for human cranial neural crest cells [26], H3K27me3, H3K4me2, H3K27ac, sequential H3K27me3/H3K4me2 and ATAC-seq signal tracks for mouse cranial neural crest cell [27] were downloaded from Gene Expression Omnibus (GEO) database [28]. Histone modification ChIP-seq data for chondrocytes and bone marrow cell were downloaded from the NCBI epigenome roadmap project (<http://www.ncbi.nlm.nih.gov/geo/roadmap/epigenomics/>). Genehancer [29] and OregAnno [30] databases were used to annotate regulatory elements regions. ENCODE transcription factor ChIP-seq tracks [31] were used to identify transcription factors binding sites.

The scores of association summary statistics for height were downloaded from the GIANT consortium ([http://www.broadinstitute.org/collaboration/giant/index.php/ GIANT_consortium_data_files](http://www.broadinstitute.org/collaboration/giant/index.php/%20GIANT_consortium_data_files)) [32]. Here we used the height GIANT and UK BioBank meta-analysis summary statistics [32] to evaluate the association between the selected genes in Cambodian aborigines and the height phenotype. We also checked the consistency of allele-specific positive and negative effects between natural selection and GWAS scores (i.e. whether the selected allele was associated with decreased height).

Haplotype networks were constructed with the median joining method [33] and visualized using NETWORK v10 (<https://www.fluxus-engineering.com/>). The geographic distribution of allele frequencies of world populations were generated with self-created R scripts.

**Reporter gene assays**

We chose PAX3-123 (rs13018600, rs12995399 and rs1367408), PAX3-4 (rs7600206), ENTPD1-1 (rs11188572), ENTPD1-2 (rs11188593), and ENTPD1-3 (rs11188612) to test their potential effect on enhancer activity by luciferase reporter assay [34]. The 500~1000bp synthetic single-strand oligonucleotides were annealed to form double strands consisting of the corresponding genotypes (“CC, TT” , “TT, CC” and “CC, TT”) for rs13018600, rs12995399 and rs1367408, (“TT, CC”) for rs7600206, “TT, AA” for rs11188572, “CC, TT” for rs11188593, and “TT, GG” for rs11188612. The oligonucleotides are flanked by restriction sites. The fragments were cloned into the multiple cloning site of the pGL3-promoter vector (Promega). All constructs of the built plasmids were validated by sequencing to make sure no de novo mutation was introduced. The ancestral alleles are “C, T and C” for rs13018600, rs12995399 and rs1367408, “T” for rs7600206, “T” for rs11188572, “C” for rs11188593, and “T” for rs11188612 (Fig. 3b and 4c). The reporter vectors containing either the ancestral allele or the derived allele were co-transfected into HEK293T and SK-N-SH cells respectively, together with a reference vector (pRL-TK vector). HEK293T and SK-N-SH cells were grown in Gibco Dulbecco’s Modified Eagle’s Medium (Gibco) supplemented with 10% fetal bovine serum (HyClone). Lipofectamine 3000 (Invitrogen) was used in transient transfection. After 36h incubation in 21% oxygen and 5% CO2, we collected the cell lysates and measured luciferase activity using the Dual-Luciferase Reporter Assay System (Promega, Madison, WI). The relative light units were measured using a luminometer. The mean values of three independent experiments were used. Each independent experiment has three replicates so that 9 data points were generated for each allele of the tested SNPs.

**References**

1. Zhang, X, Qi, X, Yang, Z*, et al.* Analysis of mitochondrial genome diversity identifies new and ancient maternal lineages in Cambodian aborigines. *Nat Commun*. 2013; **4**: 2599.

2. Zhang, X, Liao, S, Qi, X*, et al.* Y-chromosome diversity suggests southern origin and Paleolithic backwave migration of Austro-Asiatic speakers from eastern Asia to the Indian subcontinent. *Sci Rep*. 2015; **5**: 15486.

3. Schubert, M, Lindgreen, S, Orlando, L. AdapterRemoval v2: rapid adapter trimming, identification, and read merging. *BMC Res Notes*. 2016; **9**(1): 1-7.

4. Li, H, Durbin, R. Fast and accurate short read alignment with Burrows–Wheeler transform. *Bioinformatics*. 2009; **25**(14): 1754-60.

5. Manichaikul, A, Mychaleckyj, JC, Rich, SS*, et al.* Robust relationship inference in genome-wide association studies. *Bioinformatics*. 2010; **26**(22): 2867-73.

6. Consortium, GP. A global reference for human genetic variation. *Nature*. 2015; **526**(7571): 68-74.

7. Pagani, L, Lawson, DJ, Jagoda, E*, et al.* Genomic analyses inform on migration events during the peopling of Eurasia. *Nature*. 2016; **538**(7624): 238-42.

8. Mallick, S, Li, H, Lipson, M*, et al.* The Simons Genome Diversity Project: 300 genomes from 142 diverse populations. *Nature*. 2016; **538**(7624): 201-6.

9. Wong, LP, Lai, JK, Saw, WY*, et al.* Insights into the genetic structure and diversity of 38 South Asian Indians from deep whole-genome sequencing. *PLoS Genet*. 2014; **10**(5): e1004377.

10. Wong, LP, Ong, RT, Poh, WT*, et al.* Deep whole-genome sequencing of 100 southeast Asian Malays. *Am J Hum Genet*. 2013; **92**(1): 52-66.

11. Lu, D, Lou, H, Yuan, K*, et al.* Ancestral Origins and Genetic History of Tibetan Highlanders. *Am J Hum Genet*. 2016; **99**(3): 580-94.

12. Mondal, M, Casals, F, Xu, T*, et al.* Genomic analysis of Andamanese provides insights into ancient human migration into Asia and adaptation. *Nat Genet*. 2016; **48**(9): 1066-70.

13. Deng, L, Hoh, BP, Lu, D*, et al.* The population genomic landscape of human genetic structure, admixture history and local adaptation in Peninsular Malaysia. *Hum Genet*. 2014; **133**(9): 1169-85.

14. Du, Z, Ma, L, Qu, H*, et al.* Whole Genome Analyses of Chinese Population and De Novo Assembly of A Northern Han Genome. *Genomics Proteomics Bioinformatics*. 2019; **17**(3): 229-47.

15. Alexander, DH, Novembre, J, Lange, K. Fast model-based estimation of ancestry in unrelated individuals. *Genome Res*. 2009; **19**(9): 1655-64.

16. Pickrell, J, Pritchard, J. Inference of population splits and mixtures from genome-wide allele frequency data. *PLoS Genet*. 2012; **8**(11): e1002967.

17. Yi, X, Liang, Y, Huerta-Sanchez, E*, et al.* Sequencing of 50 human exomes reveals adaptation to high altitude. *Science*. 2010; **329**(5987): 75-8.

18. Cavalli-Sforza, LL. Human diversity. In: *Proc 12th Int Congr Genet,* *1969*, p. 405-16.

19. Voight, BF, Kudaravalli, S, Wen, X*, et al.* A map of recent positive selection in the human genome. *PLoS Biol*. 2006; **4**(3): e72.

20. Sabeti, PC, Varilly, P, Fry, B*, et al.* Genome-wide detection and characterization of positive selection in human populations. *Nature*. 2007; **449**(7164): 913-8.

21. Ayres, DL, Darling, A, Zwickl, DJ*, et al.* BEAGLE: an application programming interface and high-performance computing library for statistical phylogenetics. *Syst Biol*. 2012; **61**(1): 170-3.

22. Szpiech, ZA, Hernandez, RD. Selscan: an efficient multi-threaded program to perform EHH-based scans for positive selection. *Mol Biol Evol*. 2014; **31**(10): 2824-7.

23. Daub, JT, Hofer, T, Cutivet, E*, et al.* Evidence for polygenic adaptation to pathogens in the human genome. *Mol Biol Evol*. 2013; **30**(7): 1544-58.

24. Xie, C, Mao, X, Huang, J*, et al.* KOBAS 2.0: a web server for annotation and identification of enriched pathways and diseases. *Nucleic Acids Res*. 2011; **39**: W316-22.

25. Strimmer, K. fdrtool: a versatile R package for estimating local and tail area-based false discovery rates. *Bioinformatics*. 2008; **24**(12): 1461-2.

26. Prescott, SL, Srinivasan, R, Marchetto, MC*, et al.* Enhancer divergence and cis-regulatory evolution in the human and chimp neural crest. *Cell*. 2015; **163**(1): 68-83.

27. Minoux, M, Holwerda, S, Vitobello, A*, et al.* Gene bivalency at Polycomb domains regulates cranial neural crest positional identity. *Science*. 2017; **355**(6332): eaal2913.

28. Barrett, T, Wilhite, SE, Ledoux, P*, et al.* NCBI GEO: archive for functional genomics data sets--update. *Nucleic Acids Res*. 2013; **41**: D991-5.

29. Fishilevich, S, Nudel, R, Rappaport, N*, et al.* GeneHancer: genome-wide integration of enhancers and target genes in GeneCards. *Database (Oxford)*. 2017; **2017**.

30. Lesurf, R, Cotto, KC, Wang, G*, et al.* ORegAnno 3.0: a community-driven resource for curated regulatory annotation. *Nucleic Acids Res*. 2016; **44**(D1): D126-32.

31. Gerstein, MB, Kundaje, A, Hariharan, M*, et al.* Architecture of the human regulatory network derived from ENCODE data. *Nature*. 2012; **489**(7414): 91-100.

32. Loic, Y, Julia, S, E, KK*, et al.* Meta-analysis of genome-wide association studies for height and body mass index in ~700,000 individuals of European ancestry. *Hum Mol Genet*. 2018; **27**(20): 3641-9.

33. Bandelt, H-J, Forster, P, Röhl, A. Median-joining networks for inferring intraspecific phylogenies. *Mol Biol Evol*. 1999; **16**(1): 37-48.

34. Yang, D, Peng, Y, Cui, C*, et al.* HMOX2 Functions as a Modifier Gene for High‐Altitude Adaptation in Tibetans. *Hum Mutat*. 2016; **37**(2): 216-23.
